# EFFECT OF AN ORAL HEALTH PROMOTION PROGRAM ON GASTROPLASTY PATIENTS: A RANDOMIZED CLINICAL TRIAL

**DOI:** 10.1590/0102-6720202400011e1804

**Published:** 2024-06-17

**Authors:** Ilma Carla DE-SOUZA, Márjori FRÍTOLA, Valéria Campos Mariano FRANCELINO, Nathalia Maciel CORSI, Sandra Mara MACIEL

**Affiliations:** 1 Universidade Estadual de Maringá, Odontology – Maringá (PR), Brazil.

**Keywords:** Oral health, Health promotion, Obesity, Bariatric surgery, Saúde bucal, Promoção da saúde, Obesidade, Cirurgia bariátrica.

## Abstract

**BACKGROUND:**

Bariatric surgery can cause oral health problems in individuals, such as an increase in dental caries, periodontal diseases and dental erosion, which can be avoided if oral health promotion actions are implemented.

**AIMS:**

To assess the impact of an oral health promotion program implemented among gastroplasty patients.

**METHODS:**

This randomized clinical trial involved 208 patients undergoing gastroplasty; they were divided into two groups: Intervention Group, with participation in the Oral Health Promotion Program for Bariatric Patients, or Control Group. Assessments were carried out preoperatively, and six and 12 months postoperatively. The oral conditions assessed were: dental caries, periodontal diseases, tooth wear, dental plaque, and salivary flow. Sociodemographic information was obtained through application of structured questionnaires. For data analysis, the Chi-Square, Fisher’s Exact, and Mann-Whitney tests were performed — α=5%.

**RESULTS:**

Patients in the Intervention Group, when compared to those in the Control Group, presented: fewer changes in enamel (6M: p<0.0001; 12M: p=0.001), in dentin (6M: p<0.0001; 12M: p<0.0001), moderate tooth wear (6M=0.002; 12M=0.005), gingival bleeding (6M: p<0.0001), dental calculus (6M=0.002; 12M: p=0.03), periodontal pocket 4-5 mm (6M=0.001; 12M: p=<0.0001); greater reduction in the bacterial plaque index (6M: p<0.0001; 12M: p<0.0001), and increased salivary flow (6M: p=0.019).

**CONCLUSIONS:**

The oral health promotion program had a positive impact on the prevention and control of the main problems to the oral health of the gastroplasty patients.

## RESUMO

**RACIONAL:**

A cirurgia bariátrica pode ocasionar agravos bucais no indivíduo, como o aumento de cárie dentária, doença periodontal e erosão dentária, que podem ser evitados se ações de promoção em saúde bucal forem implementadas.

**OBJETIVO:**

Avaliar o impacto de um programa de promoção de saúde bucal implementado junto a gastroplastizados.

**MÉTODOS:**

Este ensaio clínico randomizado envolveu 208 pacientes submetidos à gastroplastia, divididos em dois grupos: Grupo de Intervenção, com participação no Programa de Promoção de Saúde Bucal para Pacientes Bariátricos ou Grupo Controle. As avaliações foram feitas no pré-operatório e pós-operatório de 6 e 12 meses. As condições bucais avaliadas foram: cárie dentária, doença periodontal, desgaste dentário, placa dentária e fluxo salivar. Informações sociodemográficas foram obtidas pela aplicação de questionários estruturados. Para análise dos dados foram aplicados os testes Qui- Quadrado, Exato de Fisher e Mann-Whitney, α=5%.

**RESULTADOS:**

Pacientes do Grupo de Intervenção, quando comparados aos do Grupo Controle, apresentaram: menos alterações de esmalte (6M: p<0,0001; 12M: p=0,001), de dentina (6M: p<0,0001; 12M: p<0,0001), desgaste dentário moderado (6M=0,002; 12M=0,005), sangramento gengival (6M: p<0,0001), cálculo dentário (6M=0,002; 12M: p=0,03), bolsa periodontal de 4-5 mm (6M=0,001; 12M: p=<0,0001); maior redução no índice de placa bacteriana (6M: p<0,0001; 12M: p<0,0001) e aumento do fluxo salivar (6M: p=0,019).

**CONCLUSÕES:**

Houve impacto positivo do programa de promoção de saúde bucal na prevenção e controle dos principais agravos à saúde bucal dos gastroplastizados.

## INTRODUCTION

Obesity is associated with more than 200 comorbidities, in addition to cancer, cardiovascular diseases and stroke — pathologies responsible for an increase in mortality rates worldwide^12^. Bariatric patients seek surgery to improve their life expectancy and condition, due to the negative impact that obesity and associated diseases have on their health^21,22^.

The repercussion of bariatric surgery complications on oral health has not been adequately reported in the few longitudinal studies found in the literature^20^, which justifies the need for further research providing information to promote the oral health of these patients and healthcare protocols with educational-preventive guidelines^8^.

Gastroplasty can cause nutritional deficiencies and the dumping syndrome, with nausea and vomiting, in addition to eating disorders, such as anorexia, bulimia and binge eating, which negatively affect the oral cavity^14^, causing oral problems such as periodontal disease^4^, increased dental caries^21^, dehydration and difficulty in ingesting liquids, with the consequences being hyposalivation^7^, dental erosion^14^, as well as alveolar bone loss resulting from osteoporosis, and tooth loss^23^.

Given that the determinants of oral diseases and the common risk factors for these and other chronic diseases are known^18^, oral health should be integrated into strategies aimed at promoting general health, in order to improve the living conditions of gastroplasty patients.

The alternative hypothesis of the present study is that an oral health prevention program can positively impact the oral health of bariatric patients, preventing the main oral health problems they suffer.

The objective of this randomized clinical trial was to implement and analyze the effects of an oral health promotion program among bariatric patients.

## METHODS

A controlled randomized clinical trial, registered in the Brazilian Registry of Clinical Trials (ReBEC) under No. RBR-2KCH38, received approval from the Research Ethics Committee of the Universidade Estadual de Maringa (PR), under process No. 1.113.842 and Certificate of Presentation for Ethical Appreciation (CAEE) 43114215.7.0000.0104. The study was conducted at the Obesity Surgery Center in Maringá, Paraná, and at the Diagnosis and Surgery Center in Campo Mourão (PR), between 2015 and 2021.

A total of 208 patients eligible for bariatric surgery were divided into two groups: Intervention Group (IG) and Control Group (CG). Previously, a pilot study was carried out with 30 patients to calculate the sample size; they did not participate in the research.

One sample was calculated for each outcome, and the one with the largest number was considered. A significance level of 5% and a test power of 80% were adopted, and so was a minimally significant clinical difference of 30%. Taking into account the possibility of losses in the longitudinal study, there was a 20% increase in the sample.

The inclusion criteria were age between 18 and 60 years^3^, signing of the Free and Informed Consent Form (FICF) and availability to attend the appointments. The exclusion criteria were the presence of edentulism and physical and/or mental limitations.

After opening sealed opaque envelopes containing the initials IG, for Intervention Group, and CG, for Control Group, provided by two trained nurses, before the oral health assessments, participants were randomly allocated to the IG, through which they joined the Oral Health Promotion Program for bariatric patients (PROBARI) (n=105), or to the CG, which received only the usual care from the clinic’s health team (n=103).

Clinical examinations were conducted with both the examiner and the examined person sitting, under a spotlight, with the aid of a flat mouth mirror, a World Health Organization probe (Community Periodontal Index — CPI probe)^25^, and gauze. The assessments were made by a single examiner, and information was recorded by a single notetaker, on an individualized form. A total of 10% of re-examinations were carried out to calculate the Kappa agreement index and the percentage of disagreement (clinical examination).

The oral conditions and respective indices used were: dental caries (International Caries Detection and Assessment System)^10^, periodontal disease (Community Periodontal Index)^25^, tooth wear (Tooth Wear Index) adapted by Sales-Peres et al.^16^, and dental plaque^13^. Additionally, salivary flow was measured^8^.

The PROBARI comprised instructions on dietary and oral hygiene, plaque control, salivary flow stimulation, and topical applications of fluoride varnish; the educational material was prepared from studies with educational and preventive interventions in oral health for bariatric patients, based on current scientific evidence^2,6,15^.

Before the surgical procedure, the groups (CG and IG) were given a preventive kit containing a CS 5460 toothbrush and a printed leaflet on oral health care. The instructions were reinforced monthly only for the IG patients in the postsurgical period, through Teleorientation (WhatsApp messages); the first message was sent two weeks after the educational material was delivered, and the others were sent monthly until twelve months of follow-up were completed^2,9^.

The IG patients received individual instructions regarding a less acidic and sweet diet, in order to prevent dental caries and erosion; increased water intake for greater salivary flow, and not brushing immediately after vomiting, so as to have some time between ingesting acid food and brushing the teeth, in order to inhibit the process of tooth erosion due to the association with abrasion from tooth brushing^15^.

Preventive actions included control of dental plaque and application of Clinpro White Varnish (3M ESPE) 5% fluoride varnish; they were carried out in the Obesity Surgery Centers 6 months (6M) and 12 months (12M) after the surgery, among the IG patients^5^.

**Figure 1:**
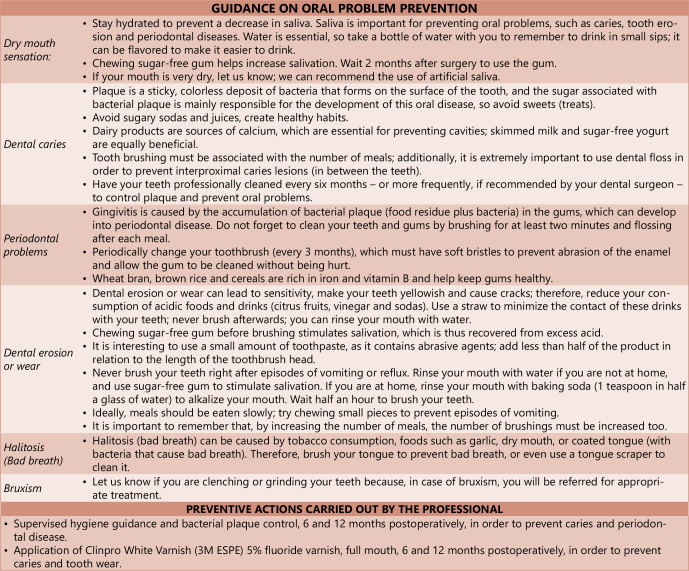
Protocol for promoting the oral health of bariatric patients.

Figure 1 presents a summary of the guidelines and preventive actions developed in the PROBARI^2,5,21^.

The Statistical Package for Social Sciences (SPSS) version 20.0 was used; through bivariate analysis, possible associations between the study variables were verified. For comparisons between the CG and IG, non-parametric statistics were used, by means of the Mann-Whitney, Chi-Square and Fisher’s Exact tests for categorical variables. The significance level adopted was 5%.

## RESULTS

Of the total number of bariatric patients who began the research, assessed preoperatively (n=208), 73.5% underwent the second assessment six months postoperatively (CG, n=103; IG, n= 105), and 50% underwent the third assessment 12 months postoperatively (CG, n=48, IG, n=56). Losses occurred because some patients, feeling well, did not return to their medical appointments.

The IG and CG groups were matched regarding sociodemographic characteristics and the type of surgery of their members (p>0.05).

Preoperatively, there was a lower bacterial plaque index (p=0.038) and lower salivary flow for the CG (p=0.003), compared to the means observed in the IG (Table 1). Six months after the surgery, a lower rate of bacterial plaque in the IG (p<0.0001), as well as a greater salivary flow (p=0.019), could be identified. At 12 months, after bariatric surgery, a statistically significant difference was observed in the salivary flow index between the groups (p=0.785).

**Table 1:** Mean and standard deviation for plaque index and salivary flow by group, in the preoperative period (n=208), and 6 months (nN=153) and 12 months (n=104) postoperatively.

Criteria	Groups		p-value
	CG	IG	
	Mean (SD)	Mean (SD)	
Preoperative plaque index	31.83 (28.3)	40.81 (31.46)	0.038
Plaque index after 6M	66.82 (29.31)	15.45 (21.97)	<0.0001
Plaque index after 12M	56.19 (34.62)	20.44 (25.65)	<0.0001
Preoperative salivary flow	1.55 (0.80)	1.59 (3.21)	0.003
Salivary flow after 6M	1.49 (0.78)	1.76 (0.77)	0.019
Salivary flow after 12M	1.89 (1.06)	1.68 (0.71)	0.785

Mann-Whitney test p<0.05. CG: Control Group; IG: Intervention Group; SD: Standard Deviation.

Comparing the CG and IG groups, in relation to periodontal conditions (Table 2), there was a significant difference for all criteria of the Community Periodontal Index (CPI) in the 6-month postoperative period, with better conditions for the IG concerning gingival bleeding (p<0.0001), dental calculus (p=0.002), 4–5mm pocket (p=0.001) and 6mm/+ pocket (p=0.031). In the 12-month postoperative period, there was a significant difference between the groups for presence of dental calculus (p= 0.003) and 4–5 mm pocket (p<0.0001), being favorable to IG.

**Table 2:** Average number of sextants affected by periodontal problems measured by the community periodontal index, in accordance with the groups and assessment periods.

Criteria	Preoperative (n=208)			After 6 months			After 12 months		
	CG	IG	p-value	CG	IG	p-value	CG	IG	p-value
	Mean (SD)	Mean (SD)		Mean (SD)	Mean (SD)		Mean (SD)	Mean (SD)	
Healthy	6.47 (3.36)	5.44 (3.67)	0.039	2.65 (3.31)	4.44 (4.32)	0.058	2.01 (2.98)	3.00 (3.97)	0.384
Bleeding	1.44 (2.42)	2.20 (3.08)	0.066	2.12 (3.05)	0.49 (1.57)	0.000	0.91 (2.00)	0.52 (1.82)	0.010
Dental calculus	0.38 (0.68)	0.41 (1.04)	0.634	0.61 (1.24)	0.21 (0.54)	0.002	0.39 (1.00)	0.10 (0.30)	0.003
4–5 mm pocket	0.77 (1.71)	0.80 (1.82)	0.865	1.38 (2.47)	0.30 (0.92)	0.001	1.61 (2.50)	0.30 (0.84)	0.000
6 mm/+ pocket	0.02 (0.13)	0.06 (0.33)	0.418	0.10 (0.40)	0.08 (0.78)	0.031	0.05 (0.33)	0.00 (0.97)	0.167

Mann-Whitney test p<0.05. CG: Control Group; IG: Intervention Group; SD: Standard Deviation.

Based on criteria of the International Caries Detection and Assessment System (ICDAS) index (Table 3), in the preoperative period, no statistical difference was identified between the groups for prevalence of caries. After six months, there was a lower incidence of changes in the IG, with a significant difference in relation to changes in both the enamel (p<0.0001) and dentin (p<0.0001). At 12 months, the result was again favorable to the IG, with a significant difference for changes in both the enamel (p=0.001) and dentin (p<0.0001).

**Table 3:** Mean and standard deviation of the International Caries Detection and Assessment System (ICDAS) index criteria, in accordance with the groups and assessment periods.

Criteria	Preoperative (n=208)			After 6 months (n=153)			After 12 months (n=104)		
	CG	IG	p-value	CG	IG	p-value	CG	IG	p-value
	Mean (SD)	Mean (SD)		Mean (SD)	Mean (SD)		Mean (SD)	Mean (SD)	
Unaltered crown	26.95 (5.14)	26.52 (4.88)	0.260	18.54 (12.15)	16.70 (13.53)	0.638	13.50 (12.91)	12.06 (13.69)	0.704
Change in enamel	0.86 (1.60)	0.86 (1.93)	0.955	1.64 (2.62)	0.41 (0.97)	<0.0001	1.38 (2.40)	0.30 (0.75)	0.001
Change in dentin	0.38 (0.91)	0.39 (0.87)	0.694	0.91 (1.72)	0.12 (0.43)	<0.0001	0.83 (1.71)	0.12 (0.49)	<0.0001
Missing teeth	3.81 (4.92)	4.23 (4.53)	0.216	10.19 (13.18)	14.76 (13.90)	0.007	16.29(14.55)	19.52 (14.07)	0.057

Mann-Whitney test p<0.05. CG: Control Group; IG: Intervention Group; SD: Standard Deviation.

In the 6-month postoperative period, with respect to the Dental Wear Index — DWI (Table 4), comparing the groups, there was a significant difference for the moderate criterion (p=0.002), being favorable to the IG. At 12 months, a lower mean for the IG could also be observed regarding the “moderate wear” criterion (p=0.005). The highest mean for severe wear was found in the CG group at 12 months, although statistical significance was not reached (p=0.092).

**Table 4:** Mean and standard deviation of the Dental Wear Index (DWI) criteria, in accordance with the groups and assessment periods.

Criteria	Preoperative (n=208)			After 6 months (n=153)			After 12 months (n=104)		
	CG	IG	p-value	IG		p-value	CG	IG	p-value
	Mean (SD)	Mean (SD)	CG	Mean (SD)	Mean (SD)		Mean (SD)	Mean (SD)	
Normal	23.27 (6.18)	20.62 (7.11)	0.040	14.34 (10.48)	12.25 (11.06)	0.170	8.80 (9.68)	8.4 (10.17)	0.626
Incipient	4.80 (4.34	6.22 (5.23)	0.052	5.77 (6.40)	4.75 (5.77)	0.201	5.19 (6.61)	3.6 (5.07)	0.101
Moderate	0.14 (0.54)	0.67 (1.98)	0.029	0.90 (1.99)	0.17 (0.59)	0.002	1.27 (2.95)	0.28 (1.03)	0.005
Severe	0.00 (0.00)	0.01 (0.09)	0.332	0.00 (0.00)	0.00 (0.00)	1.000	0.20 (1.25)	0.01 (0.98)	0.092
Restored	-	-	-	-	-	-	-	-	-

Mann-Whitney test p<0.05. CG: Control Group; IG: Intervention Group; SD: Standard Deviation.

## DISCUSSION

To date, we believe that this is the first randomized clinical trial to describe the impact of a health promotion program on the oral conditions of gastroplasty patients, with a 12-month longitudinal follow-up.

After gastroplasty (gastric sleeve and gastric bypass), oral health indicators, assessed after six and 12 months, showed better conditions for IG patients, with a significant reduction in oral health problems such as dental caries, periodontal disease, dental plaque and xerostomia, compared to the CG.

Corroborating a previous study^21^, which highlighted a high amount of bacterial plaque in gastroplasty patients, in this study, CG patients showed greater accumulation of plaque after six and 12 months. On the other hand, patients in the IG had a significant reduction in plaque index at both six and 12 months.

The present study is in line with research that also recommends periodic prophylaxis to help improve the oral hygiene status of patients undergoing bariatric surgery, which results in the prevention of oral diseases such as periodontitis, thus greatly improving the patient’s quality of life^20^.

In a study by Elward et al.^5^, patients reported water intolerance, caused by gastric changes resulting from the surgery. To help with their intake and improve salivary flow, IG patients were advised to increase their water consumption by carrying a bottle of flavored water with them, in order to facilitate ingestion in small sips, in addition to using sugar-free gum in order to stimulate salivation, or using artificial saliva, when the patient had xerostomia^14^.

Although preoperatively most patients with xerostomia were part of the IG, possibly due to the use of xerostomia drugs^11^, this group achieved a significant increase in their average salivary flow in the 6-month postoperative period compared to the CG. After 12 months, corroborating the report by Quintella et al.^15^, no significant difference was observed between the groups, given that normalization of salivary flow was expected.

An increase in gingival bleeding, peaking at six months, is expected after bariatric surgery^4^; in this study, while patients in the CG showed an increase in gingival bleeding six months after surgery, participants in the IG promotion program showed a significant reduction (p<0.0001), a fact that may be related to the oral health promotion actions that were developed in this group. In the 12-month postoperative period, there was also a significant difference between the groups for presence of dental calculus (p=0.002) and 4–5mm periodontal pocket (p<0.0001), with the results being favorable to the IG.

After six and 12 months, the incidence of caries was significantly lower among members of the IG compared to those in the CG, both in enamel and dentin. A previous study, in which there was no dental intervention, reported a significant increase in dental caries over time following gastroplasty^20^.

The bariatric diet, combined with inadequate oral hygiene, may result in an increase in carious lesions, because, with reduced gastric capacity, there is a medical recommendation to increase the frequency of food and liquid intake throughout the day^20^. Furthermore, hyposalivation, common in gastroplasty patients in the first months after surgery, is accompanied by an increase in cariogenic microorganisms, such as *Lactobacillus* and *Streptococcus mutans*, increasing the risk of caries, especially in the first six months^7^.

Topical applications of fluoride varnish on all dental surfaces, every six months^24^, as well as educational-preventive guidance to improve hygiene, diet and salivary flow, had good results, such as the significant prevention of changes in enamel and dentin, and of tooth wear, in the two assessment periods (six and 12 months) of this study. The effectiveness of the product is due to its action in preventing and remineralizing carious lesions, in addition to preventing tooth erosion and cases of dentin hypersensitivity^19^.

A clinical trial conducted by Azevedo et al.^1^ found a higher prevalence of tooth wear in patients after bariatric surgery; however, there was a lower occurrence of moderate tooth wear at six and 12 months for the IG, with a significant difference between the groups.

Corroborating a randomized clinical trial conducted by Scheerman et al.^17^, which reports that usual care combined with the use of a mobile application to send patients preventive messages provides oral health education, the findings of this study also showed the effectiveness of oral health education carried out by dental surgeons, based on theory and approach, with a significant improvement in patients’ oral hygiene behavior^17^.

The patients in both groups had to be warned about the need for extractions, which could harm their recovery after gastroplasty if not performed, this being a possible limitation for the study, given that tooth losses were recorded also in the IG. Furthermore, not all patients returned to the clinic until the end of the research, since, if they felt well, they did not return to the multidisciplinary team appointments, which reduced the sample size in the second and third assessments (six and 12 months after surgery).

The educational-preventive care protocol narrated and evaluated in this trial proved to be effective in promoting the oral health of bariatric patients, preventing the impact of bariatric surgery on oral health in the IG, being significant for: salivary flow and gingival bleeding at six months, with significant results six and 12 months after surgery; changes in enamel and dentin, dental calculus and 4–5mm periodontal pocket, a reduction in the bacterial plaque index, in addition to less tooth wear rated as moderate.

Further research, with a longer follow-up period, is necessary in order to assess the greater benefits that the oral health promotion program can provide, over time, to gastroplasty patients.

## CONCLUSIONS

The oral health promotion program had a positive impact on the prevention of dental caries, periodontal disease, xerostomia, dental erosion and plaque accumulation in gastroplasty patients, which reinforces the importance of including dental surgeons in the multidisciplinary teams that assist them. REFERENCES
